# Gene polymorphism of interleukin 1 and 8 in chronic gastritis patients infected with *Helicobacter pylori*

**DOI:** 10.1186/1678-9199-20-17

**Published:** 2014-04-23

**Authors:** Agostinho Caleman Neto, Lucas T Rasmussen, Roger W de Labio, Valdeir F de Queiroz, Marília de AC Smith, Gustavo A Viani, Spencer LM Payão

**Affiliations:** 1 Department of Genetics, FAMEMA Blood Center, Marília Medical School (FAMEMA), Marília, São Paulo State, Brazil; 2 Sacred Heart University (USC), Bauru, São Paulo State, Brazil; 3 Department of Digestive System Surgery, Marília Medical School (FAMEMA), Marília, São Paulo State, Brazil; 4 Department of Morphology, Federal University of São Paulo (UNIFESP), São Paulo, São Paulo State, Brazil; 5 Department of Radiotherapy and Oncology, Marília Medical School (FAMEMA), Marília, São Paulo State, Brazil; 6 Laboratório de Genética, Hemocentro, FAMEMA, Rua Lourival Freire, 240, Bairro Fragata, Marília, São Paulo CEP 17519-050, Brazil

**Keywords:** *Helicobacter pylori*, Polymorphism, Interleukin

## Abstract

**Background:**

Epidemiological investigations have indicated that *Helicobacter pylori* induces inflammation in the gastric mucosa regulated by several interleukins. The genes *IL1B* and *IL8* are suggested as key factors in determining the risk of gastritis. The aim of this paper was to evaluate the association of gene polymorphism of interleukin-1 and interleukin-8 with chronic gastrits in *H. pylori* infected patients. A total of 60 patients underwent endoscopic procedure. Biopsy samples were collected for urease test, histopathological and molecular exams. The DNA of theses samples was extracted for detection of *H. pylori* and analysis of the genes mentioned above. Patients with gastritis had a higher frequency of *H. pylori*-positive samples.

**Results:**

*H. pylori* was detected in 30/60 patients (50%) by PCR. As for polymorphism of interleukin 8 (-251) gene we observed a statistical difference when analyzed TA (*p* = 0.039) and TT (*p* = 0.047) genotypes. In the *IL1B*31 there was a statistical difference in TT (*p* = 0.01) genotype and in the *IL1B-511* there wasn’t any statistical difference.

**Conclusion:**

Our results suggest a strong correlation between the presence of chronic gastritis and infection by *H. pylori* and that *IL1B-31TT* and *IL8-251TT* genotypes appear to act as protective factors against *H. pylori* infection while *IL8-251TA* genotype may comprise a risk factor for infection with this bacterium.

## Background

*Helicobacter pylori* is a gram-negative bacterium that colonizes the gastric antrum and/or the body of the human stomach, causing complications such as gastritis, gastric and duodenal ulcer and gastric malignancies [1,2]. *H. pylori* is present in approximately half of the world population [3]. Numerous studies have shown a significant relationship between the bacteria and the risk of gastritis [4,5]. Atherton [6] reported that the development of gastric diseases is influenced by the degree of virulence of the *H. pylori* strain, the host genetic susceptibility and environmental cofactors.

Some virulence factors, such as *vacA* and *cagA* genes, were studied since they could play an important role in the pathogenesis of infection with *H. pylori*i. The *vacA* gene, present in all strains, covers two different regions, the “s” region (s1 and s2) and the “m” region (m1 and m2) [7]. Another relevant marker of pathogenicity is the *cagA* gene, present in about 60-70% of the *H. pylori* strains. Infection with *H. pylori cagA* + strains have been associated with a greater degree of inflammation of the gastric mucosa and severe atrophic gastritis. Therefore, it has been suggested to play an important role in the development of gastric carcinoma [7,8].

Chronic infection with *H. pylori* results in inflammation of the gastric mucosa, which induces the expression of proinflammatory cytokines such as interleukins, tumor necrosis factor (alpha), and interferon [9].

Several genetic polymorphisms related to inflammatory cytotoxins have been studied and associated with an increase in the synthesis of these interleukins. The polymorphisms of some interleukins are important in cancer susceptibility [10,11]. *H. pylori* may indirectly stimulate the activation of an inflammatory cascade of cytotoxins, which induces the production of chemokines for neutrophils and mononuclear cells, as well as interleukins IL8 and IL1, leading to a response with an inflammatory infiltrate and development of chronic gastritis [12]. The *IL8* gene has been described as having a polymorphism of an A/T base pair in the promoting region (-251) which is associated with an increase in the synthesis of interleukin by gastric epithelial cells [13,14].

The presence of *-31CC* or -*511TT* genotypes of interleukin *1B* may render patients at increased risk of developing gastric atrophy and hypochlorhydria by infection of *H. pylori*, which can also lead to a two- to threefold increased risk of malignancy compared to the presence of less inflammatory genotypes [15,16]. Cytokine *IL1B-31CC*/*-511TT* gene polymorphisms have been shown to be related to gastric cancer and chronic gastritis development in patients infected by *H. pylori*. [16,17]. Therefore, the aim of this paper was to evaluate association of gene polymorphisms of interleukin 1 and 8 with chronic gastrits in *H. pylori* infected patients.

## Methods

### Patients and samples

Thirty adults (mean age 49.5 years old) that tested positive for *H. pylori* infection and had recurrent abdominal pain were included in the present study. Control group comprised 30 individuals in the same age group. All subjects were recruited from the Endoscopy Unit of Marilia Medical School, SP, Brazil. Demographics of these patients were obtained from medical records. Three biopsy samples from each patient were collected from the gastric antrum in order to be submitted for histopathological examination, rapid urease test and DNA extraction.

All patients or legal guardians were informed about the objectives and the research protocol of the present study. They all signed an informed consent form approved by the Research Ethics Committee of Marília Medical School (protocol n. 1119/11).

### DNA extraction and *H. pylori* detection

DNA was extracted from the gastric biopsies using the QIAamp® tissue kit (Qiagen, Germany) according to the manufacturer’s instructions. For the detection of *H. pylori* the PCR technique was employed, which amplifies a fragment of 150 bp from the 16S rRNA of *H. pylori*[18]. PCR conditions comprised 40 cycles of amplification, each cycle consisted of 45 seconds of denaturation at 94°C, 45 seconds of annealing at 59°C and 45 seconds extension at 72°C. In each experiment, positive (strain 26695) and negative (water) controls were included.

### Histopathology and rapid urease test

The fragments were fixed with 10% formalin and stained by hematoxylin-eosin and Giemsa. Microscopic examination defined the degree of involvement of the gastric mucosa and presence of *Helicobacter pylori*.

An antral biopsy from each patient was incubated in premade broth (TUPF; Laborclin, Brazil) for the urease test immediately after collection. The test was considered positive when the color of the solution changed from yellow to orange, pink, or purple within four hours of incubation at 25°C.

### Interleukin-1β genotyping

In order to characterize *IL1β* (-31,-511) gene polymorphism, restriction fragment length polymorphism (RFLP) was employed. Similarly, polymorphism of the promoting region (-511) in the *IL1β* gene was identified using PCR primers under the conditions described by Wilkinson *et al.*[19]. *AvaI* enzyme was used to digest the PCR products at 37°C overnight. Electrophoresis in 3% agarose gel stained with ethidium bromide showed the following bands: CC (80 and 109 bp), CT (80, 109 and 189 bp) and TT (189 bp).

To the promoting region (-31) of the *IL1β* gene the *AluI* enzyme was used for digestion at 37ºC overnight [15]. The following bands, separated by electrophoresis in 3% agarose gel stained with ethidium bromide, were observed: CC (240 bp); CT ( 98, 137 and 240 bp) and TT (98 and 137 bp) [20].

### Interleukin-8 genotyping

*IL8* (-251) gene polymorphisms were characterized through RFLP as previously described [21]. The PCR product, a 349-bp fragment, was digested by *MunI* restriction enzyme (MBI Fermentas, Canada) overnight at 37°C and then separated by electrophoresis in 3% agarose gel stained with ethidium bromide. The digestion fragments had 349 bp with TT genotype, 202 and 147 bp with AA genotype and 349, 202 and 147 bp with TA genotype.

## Results

*Helicobacter pylori* was detected in 30 of the 60 patients (50%) by PCR whereas the urease test detected the bacterium in 21 patients (35%) (Table 1). All the samples in which the histology and urease test demonstrated the presence of *H. pylori* were also positive by PCR, showing a sensitivity of 65% and specificity of 100% by PCR and sensitivity of 43.5% and specificity of 92.9% by rapid urease test. The histopathological analysis revealed the presence of chronic gastritis in 46 subjects (76.6%).

**Table 1:** **PCR and urease test detection for *H. pylori *** **in patients with chronic gastritis and negative control group**

	**Chronic gastritis**		
**PCR**	**Negative (%)**	**Positive (%)**	**Total**
Positive	0 (0)	30 (100)*	30
Negative	14 (46.7)	16 (53.3)	30
**Urease**	**Negative**	**Positive**	**Total**
Positive	1 (4.8)	20 (95.2)*	21
Negative	13 (33.3)	26 (66.7)	39

*Statistical difference was observed.

In relation to the distribution of *IL1B-511* polymorphisms, no significant difference was observed in genotype distribution among patients infected with *H. pylori* and the control group. However, when polymorphism distribution of genotype *IL1B-31* was analyzed, a significant difference was found. Frequency of TT genotype was lower in *H. pylori*-infected patients (6.7%) when compared with the non-infected group (33.3%, p = 0.01).

When *IL8-251* polymorphisms were analyzed, a significant variation was found. The distribution of TA genotype was higher in *H. pylori*-infected patients (63.3%) than in the control group (36.7%, p = 0.039) whereas the frequency of TT genotype was higher in controls (43.3%) than in infected patients (20%, p = 0.047). These results of polymorphisms are displayed in Table 2.

**Table 2:** **Genotype distribution of single nucleotide polymorphisms of *IL1B *** **and *IL8 *** **in Brazilian adult patients according to PCR results for *H. pylori *** **infection**

**PCR**	***IL1B-31***				***IL1B-511***				***IL8-251***			
	**CC (%)**	**TC (%)**	TT (%)*	**Total**	**CC (%)**	**TC (%)**	**TT (%)**	**Total**	**AA (%)**	TA (%)*	TT (%)*	**Total**
**Pos.**	10 (33.3)	18 (60)	2 (6.7)	30	11 (36.7)	13 (43.3)	6 (20)	30	5 (16.7)	19 (63.3)	6 (20)	30
**Neg.**	5 (16.7)	15 (50)	10 (33.3)	30	10 (33.3)	16 (53.3)	4 (13.4)	30	6 (20)	11 (36.7)	13 (43.3)	30

*Statistical difference was observed.

## Discussion

In the present study, we analyzed gene polymorphisms of *IL8* and *IL1B* together with *H. pylori* infection in groups of adult patients from Marília, SP, Brazil. Recently, researchers have been focusing their attention on the possible connection between human gene polymorphisms and *H. pylori* infection. From the investigated genes, it is important to emphasize the role of proinflammatory IL-1 polymorphism (*IL1B-511 T*/*-31C*), demonstrated in experiments conducted by El-Omar *et al*. [15].

In the Japanese population, *IL1B-511C* > *C* polymorphism was dominant among patients with advanced atrophic chronic gastritis, whereas *IL1B-511 T* > *T + T* > *C* polymorphism was more frequent in the Chinese population. In addition, no differences were found in the frequency of occurrence of C and T alleles in Tai and Vietnamese populations [22]. Regarding the Chinese case, interesting observations have shown that the *IL1B-511TT* genotype disclosed an association between peptic disease and *H. pylori*[23]. Santos *et al*. [24] found that in the Brazilian population, *IL1β-511CC* and CT gene polymorphisms were associated with chronic gastritis and gastric cancer development in *H. pylori*-infected individuals. Our results of patients non-infected and infected with *H. pylori* revealed no association with *IL1B-511* polymorphisms.

The Chinese research also found that *IL1B-31CC* genotype was more frequent in carcinoma patients than in the control group in northern China (a region characterized by high incidence of gastric carcinoma), whereas in southern China (a region with low gastric carcinoma incidence) it was more frequent in controls than in cancer patients [23]. This indicates that the T allele could act as a proinflammatory allele in genotype *IL1B-31 T* and both genotypes may constitute independent gastric carcinoma risk factors. These hypotheses were corroborated by investigations carried out in Korea, which indicated the importance of *H. pylori* infection and presence of *IL1B-31 T* and *IL1B-511C* polymorphism for the increase of *IL1B* production by the gastric mucosa [25]. Our results suggest that the genotype *IL1B-31TT* emerge as a possible protector factor against *H. pylori.* Similar results were found in Turkish patients [26].

Cheng *et al*. [27] found no association of polymorphisms of *IL8-251 T > A* with increased risk of gastritis in Thai patients, as well as Fabris *et al*. [28] who did not find any significant association between *IL-8-251 T > A* polymorphism and *H. pylori* infection in Brazilian patients. However, some studies reported controversial results. Hofner *et al*. [29] described an association between IL8 (TA) genotype and risk for gastritis or duodenal ulcers in patients infected with *H. pylori*. Ohyauchi *et al*. [11] and Taguchi *et al*. [13] observed that the AA genotype confers higher risk for atrophic gastritis compared to the TT genotype in patients with *H. pylori*. These authors also associated TA and AA genotypes with augmented levels of IL8 and higher degree of neutrophil infiltration when compared with the TT genotype.

In the present study, we found a significant difference between TA and TT genotypes of *IL8* and the presence of *H. pylori*. However, such relation was not true for the AA genotype, which suggests that the presence of TA genotype may be a risk factor *H. pylori* infection whereas TT genotype may act as a protector factor against *H. pylori*. In most of the eastern population the presence of the A allele in the promoting region (-251) of the *IL8* gene was related to an increased risk of stomach malignancy [11,13,30]. According to some studies, production of IL8 is augmented by the presence of A allele, and the quality and intensity of inflammatory responses produced by the host can be altered after exposure to *H. pylori*[13,14,30].

The discrepancy among *IL8* genotypes may be related to genetic differences in populations and sample sizes. However, the frequency of *IL-8-251 TA* genotype was found to be different among ethnic groups as reported in the single nucleotide polymorphism database (http://www.ncbi.nlm.nih.gov/SNP/snp_ref.cgi?rs=4073).

Polymorphisms of *IL8* are biologically important in the pathogenesis of gastric diseases. Therefore, further studies in different populations are required, since these polymorphisms in some demographic regions appear to be essential in the development of gastric diseases. Our results showed a significant role of polymorphisms of genes *IL1* and *IL8* in relation to the infection by *H. pylori* and the development of gastric mucosa inflammation. It is necessary to bear in mind that gene polymorphisms of other cytokines and other genetic factors may exert a synergistic action in the development of such changes related to *H. pylori*.

## Conclusion

In summary, the current results suggest a strong correlation between the presence of chronic gastritis and infection by *H. pylori*. In addition, the *IL1B-31TT* and *IL8-251 TT* genotypes seem to act as protective factors against *H. pylori* infection while *IL8-251TA* genotype may be a risk factor for infection with this bacterium. These polymorphisms are biologically important in the pathogenesis of gastric diseases and require more studies in different populations, since they appear to be an important variable in the development of gastric diseases in some individuals exposed to *H. pylori*.

### Ethics committee approval

Patients or legal guardians were informed about the objectives and the research protocol of the present study. They all signed an informed consent form approved by the Research Ethics Committee of Marília Medical School (protocol n. 1119/11).

## Competing interests

The authors declare that they have no competing interests.

## Authors’ contributions

All authors read and approved the final manuscript. ACN carried out the sample collection, DNA extraction and molecular analysis. RWL, LTR, GV, VFQ, MACS, SLMP and ACN participated in the study design, performed the statistical analysis and provided technical support and scientific discussions. SLMP and ACN designed of the study, coordinated the research and helped to draft the manuscript.
